# Dynamical memristive neural networks and associative self-learning architectures using biomimetic devices

**DOI:** 10.3389/fnins.2023.1153183

**Published:** 2023-04-20

**Authors:** Bill Zivasatienraj, W. Alan Doolittle

**Affiliations:** Department of Electrical and Computer Engineering, Georgia Institute of Technology, Atlanta, GA, United States

**Keywords:** neural, network, memristor, neuromorphic, learning, computation, temporal, dynamic

## Abstract

While there is an abundance of research on neural networks that are “inspired” by the brain, few mimic the critical temporal compute features that allow the brain to efficiently perform complex computations. Even fewer methods emulate the heterogeneity of learning produced by biological neurons. Memory devices, such as memristors, are also investigated for their potential to implement neuronal functions in electronic hardware. However, memristors in computing architectures typically operate as non-volatile memories, either as storage or as the weights in a multiply-and-accumulate function that requires direct access to manipulate memristance *via* a costly learning algorithm. Hence, the integration of memristors into architectures as time-dependent computational units is studied, starting with the development of a compact and versatile mathematical model that is capable of emulating flux-linkage controlled analog (FLCA) memristors and their unique temporal characteristics. The proposed model, which is validated against experimental FLCA Li_x_NbO_2_ intercalation devices, is used to create memristive circuits that mimic neuronal behavior such as desensitization, paired-pulse facilitation, and spike-timing-dependent plasticity. The model is used to demonstrate building blocks of biomimetic learning *via* dynamical memristive circuits that implement biomimetic learning rules in a self-training neural network, with dynamical memristive weights that are capable of associative lifelong learning. Successful training of the dynamical memristive neural network to perform image classification of handwritten digits is shown, including lifelong learning by having the dynamical memristive network relearn different characters in succession. An analog computing architecture that learns to associate input-to-input correlations is also introduced, with examples demonstrating image classification and pattern recognition without convolution. The biomimetic functions shown in this paper result from fully ion-driven memristive circuits devoid of integrating capacitors and thus are instructive for exploiting the immense potential of memristive technology for neuromorphic computation in hardware and allowing a common architecture to be applied to a wide range of learning rules, including STDP, magnitude, frequency, and pulse shape among others, to enable an inorganic implementation of the complex heterogeneity of biological neural systems.

## 1. Introduction

The von Neumann architecture has brought waves of advancement in computational technology for much of the past century, and record-breaking improvements have been driven according to Moore’s law. However, the foreseeable end of Moore’s law has invoked widespread research on alternatives to the von Neumann computer architecture still being used today (Theis and Wong, 2017). Neuromorphic computing describes a non-von Neumann architecture with an information-processing system inspired by the mammalian brain (Mead, 1990), which utilizes massive parallelism that bypasses the speed and efficiency limitations of CMOS devices (Haron and Hamdioui, 2008). While the definition of a memristor has been widened to include other resistive devices, the memristor was originally defined as a resistive memory device with a conductance that changes depending on the input charge or flux-linkage (Chua, 1971). Thus, the memristor is a biomimetic device capable of in-memory computation and can have biologically realistic temporal dynamics that have been rarely exploited (Zivasatienraj et al., 2020), making the memristor a promising solution to the realization of neuromorphic computing.

Memristors are being widely investigated to implement neural networks in hardware. By utilizing the energy-efficient memory to store synaptic weights, memristive neural networks can be trained to perform complex computations such as image classification. Memristors are typically assembled into a resistive crossbar array (RCA) to perform multiply-and-accumulate (MAC) operations in hardware (Chen et al., 2021). However, the RCA is still restricted by the von Neumann bottleneck due to the circuitry required to select individual memristors and avoid sneak paths (Cassuto et al., 2013). A key limitation in the crossbar architecture is the need to individually address and program each individual memristor, costing compute time and energy and functions in a non-biological manner. Biology never programs an individual synapse. Instead, signals are sent to a network of synapses and the temporal-magnitude relationships of the collective synapses program the synaptic weights, often resulting in weight dynamics that last well after the signal has passed. This temporal-magnitude biological relationship is directly analogous to flux-linkage dependent memristor behavior. In addition, backpropagation (BP) is traditionally used to program the memristive weights in the RCA, where a loss function is calculated and then used to address errors for each individual weight in the network. While being the dominant method for neural computation, BP and all methods of directly changing each and every memristor weight in a prescribed manner as defined by an algorithm is inherently non-biomimetic. Additionally, in hardware implementations, BP necessitates additional circuitry for signal processing, adding even more power consumption and latency to the system (Wilamowski, 2009). Thus, research into alternative architectures for RCAs and the costly BP process that requires direct access to specific neural weights is warranted.

Synaptic plasticity such as paired-pulse facilitation (PPF), where rapid consecutive stimuli invoke stronger neuronal responses (López, 2001), and spike-timing-dependent plasticity (STDP), where temporal correlations between stimuli affect the direction and magnitude of synaptic weight changes (Markram et al., 2012), can be biomimetic alternatives to BP by updating memristive weights *via* associative self-learning and puts the action of training into the signal timing instead of direct memristance manipulation. The application of Hebb’s postulate could allow memristive weights to train themselves without computing loss functions, relying instead on the temporal information within the timing of stimuli, and thus enabling energy-efficient and lifelong learning. Therefore, synaptic devices have shown great promise as a solution for implementing neuromorphic computing (Kwak et al., 2022). Temporally dynamic memristors with analog behavior, such as FLCA Li_x_NbO_2_ intercalation devices (Zivasatienraj et al., 2020), are well suited to implement PPF and STDP in memristive circuitry and can be utilized for diverse learning rules, including frequency, phase, magnitude, and pulse shape-based learning, to emulate the diverse heterogeneity of neural signals and learning in nature. We focus on Li_x_NbO_2_ memristors primarily because these devices have shown the ability to enable diverse engineering design options, including the static resistance (10 ohms to 10 Megaohms), the dynamic memresistance range (~90:1), and even static (~1,000x) and dynamic tuning (~10x) of the temporal response times, all by lithographic geometry configuration and Li ion intercalation with millivolt scale programing sensitivity comparable to biological neural systems (Zivasatienraj et al., 2020; Ghosh et al., 2023). Crucially, these devices can be stimulated bipolarly to change memristance up or down without having to be reset a device, making them near-ideal ion integration, and thus displacement flux, storage elements.

Creating a practical memristor model that can be used in the design and analysis of circuit-level applications would accelerate the progression of memristive technology, integration of memristors into conventional circuitry, and investigation into novel computing architectures. An extraordinary number of proposed models exist, but many are derivatives of a few fundamental models (Strukov et al., 2008; Biolek et al., 2009; Joglekar and Wolf, 2009; Pickett et al., 2009; Prodromakis et al., 2011; Yakopcic et al., 2011; Kvatinsky et al., 2013) that are adapted to be applicable for specific device technologies. However, most of these models fail to capture the analog and temporal nature of some technologies, intercalation devices included, which can continuously integrate voltage pulses or even respond to DC biases. Additionally, although able to accurately and stably simulate circuits where the memristor interacts with other non-memristor devices, such as in RCAs emulating convolutional neural networks, when many of these models are used in networks where multiple memristors are connected together, such as in reservoir or recurrent neural networks, these models often fail to converge or due to internal complexity, require enormous simulation times, particularly when the network is sizable.

It is thus desirable for memristor models to be compact and efficient for the simulation of large circuits so that practical learning applications can be studied (Biolek et al., 2018). Memristor models should have the ability to model experimental devices and address the associated non-linearities and asymmetries in device performance, including unique features such as temporal responsivity that could present new levels of complexity in computation (Zivasatienraj et al., 2020). Herein, a minimally complex yet versatile, compact memristor model is introduced that is capable of emulating various memristive mechanisms while implementing temporal dynamics, such as in neuromorphic circuitry that mimic biological functions like desensitization, PPF, and STDP. These biomimetic functions are then used to implement self-training memristive neural networks and analog computing architectures that can perform image classification of the Extended Modified National Institute of Standards and Technology (EMNIST) dataset without using expensive learning algorithms.

## 2. Materials and methods

### 2.1. Model equations

To achieve the goal of a minimally complex model capable of rapid convergence in large scale simulations, the current–voltage relation of an analog flux-controlled memristor is adapted from Chua’s original work (Chua, 1971) to be:


(1) $$i\left(t\right)=v\left(t\right)/M\left(\Phi \left(t\right)\right)$$

where $M\left(\Phi \left(t\right)\right)$, the memristance, is empirically defined as


(2) $$M\left(\Phi \left(t\right)\right)=\sum_{0}^{N}R_{n}{\left|\Phi \right|}^{n}$$

where the ${R_{n}}^{\prime }s$ are “resistance” related fitting parameters with units of $\mathrm{Coulomb}s^{-n}$ chosen both for simplicity to aid convergence as well as for maximum diversity to widen impact for device agnosticism. In this work, the minimal number of $R_{n}$ parameters are used to sufficiently fit our device and undefined $R_{n}$ parameters are assumed to be equal to zero. $\Phi \left(t\right)$ is the effective flux linkage which in its simplest form is $\Phi \left(t\right)=\int_{-\infty }^{t}v\left(\tau \right)d\tau$, which in the case of Li_x_NbO_2_ is ion accumulation in a metallic electrode (Zivasatienraj et al., 2020). For practicality, $\Phi \left(t\right)$ is calculated with an extra term to account for ion redistribution/relaxation diffusion effects in the intercalation memristor, which by analogy can be any internal hidden variable such as heat, spin polarity, charge, or ferroelectric/magnetic domain growth, that accumulates and diffuses to change device resistance (Shank, 2016). $\Phi \left(t\right)$ is iteratively calculated from $\Phi^{\prime }\left(t\right)$ by:


(3) $$\Phi^{\prime }\left(t\right)=Av\left(t\right)-D\Phi \left(t\right)$$

where *A* is a unitless gain parameter for adjusting the sensitivity to stimulus. The parameter *D* controls the (diffusive for the intercalation device case) temporal response with units of $s^{-1}$, introducing an exponentially decaying transient to the system for controllable non-linearity. In the Li_x_NbO_2_ case, parameters A and D emulate the device properties controlled by lithographically defined device size/geometry and intercalation state (Zivasatienraj et al., 2020; Ghosh et al., 2023). For a given $\Phi \left(t\right)$, $M\left(\Phi \left(t\right)\right)$ is the incremental memresistance, which specifies the resistance of the memristor depending on the accumulated flux-linkage. Together, Eqs. (1)–(3) provide a versatile model for flux-controlled analog memristors with tunable parameters for volatility, non-linearity, dynamic range, temporal response, and sensitivity to stimulus.

The flux-linkage controlled model also allows for non-volatile memristor behavior by completely removing the ion recovery effects ($D=0\;s^{-1}$). Without any subtractive (D) term, all changes to flux-linkage – and therefore resistance – would only be alterable by applied voltage and is persistent upon removal of the bias. However, Eqs. (1)–(3) do not adequately describe experimental non-volatile devices due to the missing voltage-dependent flux-linkage saturation characteristic wherein the resistance tends to saturate to different values depending on the magnitude of the applied voltage (Zivasatienraj et al., 2020). Equations (1)–(3) alone suggest that any applied voltage would be integrated into the flux-linkage regardless of current state creating a problem analogous to integral windup in PID control theory where flux-linkage grows unconstrained for an applied steady state bias. Real devices have physical limits on the flux-linkage, for example from the maximum density of ions that can be intercalated. Thus, limitations are added on the calculation of flux-linkage that enable non-volatility regardless of parameter *D*, allowing for voltage-dependent flux-linkage saturation as well as control of the temporal response from a non-volatile memristor. For non-volatile memristors, $\Phi^{\prime }\left(t\right)$ is defined to be


(4) $$\Phi^{\prime }(t)=\;\left\{ \begin{array}{cc} 0 & 0<Av(t)/D<\Phi (t)\\ Av(t)-D\Phi (t) & \mathrm{otherwise}\text{.} \end{array}\right.$$

The exponential relaxation from parameter *D* eventually negates the incorporation of a constant input voltage to flux-linkage, saturating to $\Phi \left(t\to \infty \right)=AV/D$. Therefore, the conditional limitations allow for voltage-dependent flux-linkage saturation and non-volatile voltage-dependent memristance as observed in experiments (Zivasatienraj et al., 2020). Additionally, parameter *D* tunes the asymmetry in programming polarity often observed in physical devices, as negative voltage inputs decrease the flux-linkage faster than positive voltages increase the flux-linkage. In Li_x_NbO_2_ devices, this may arise when the applied voltage opposes the electric field created by intercalated charge. In the negative input case, drift and diffusion work constructively whereas for positive inputs, drift and diffusion oppose each other. Furthermore, $\Phi \left(t\right)$ can be bounded to non-negative (as are the non-volatile memristors shown later) or arbitrary values for asymmetric operation.

Since $\Phi \left(t\right)$ has both voltage and time integrated aspects, the proposed model can account for either or both computational schemes. Specifically, like most memristor models, the voltage can change the resistance and thus be the source of computation. But with temporal dynamics explicitly controlled and extending over large time scales (not merely pico/nanoseconds), the proposed model can also use time as an analog computational variable and accurately accounts for the extended time-dependent computational capabilities of real Li_x_NbO_2_ devices. Thus, Eqs. (1)–(4) form a versatile memristor model capable of reproducing the behavior of volatile and non-volatile experimental memristors spanning wide temporal integration times and resistance dynamic ranges. This wide temporal “memory window” is critical for the implementation of frequency, phase, magnitude, and pulse shaped learning rules, including the synaptic plasticity for self-training neural networks demonstrated here.

### 2.2. Model implementation

**Figure 1:**
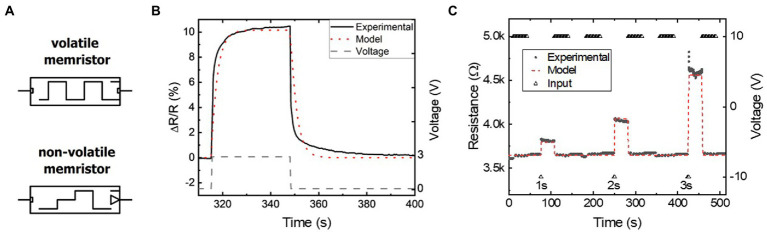
**(A)** Circuit symbols for the volatile (top) and non-volatile (bottom) memristor packaged in SPICE. **(B,C)** Parameter extraction used to match experimental **(B)** volatile and **(C)** non-volatile memristors and their time-dependent characteristics.

A memristor model implementing Eqs. (1)–(4) was built using a simulation program for integrated circuits emphasis (SPICE). The SPICE environment was chosen to emulate our FLCA memristors for seamless integration into electronic circuitry, while exploiting the dynamic time stepping built into SPICE for fast and efficient simulation. SPICE also corroborates the later discussed transition to PyTorch implementations of memristive neural networks and computing architectures. As shown in Figure 1A, separate compact SPICE component models are created for volatile and non-volatile memristors and validated *via* parameter extraction of experimental Li_x_NbO_2_ intercalation devices (Supplementary Figures 1–3; Zivasatienraj et al., 2020). The volatile decay of resistance as a function of time in Figure 1B is closely emulated by the model. Likewise, Figure 1C shows that the non-volatile resistance changes as a function of the pulse duration are replicated by the flux-linkage controlled model. Higher order Diffusive and Gain terms in Eq. (2) could be used for further accuracy at the cost of complexity and simulation time. Simulations of these devices converge equally as fast as a level 4 MOSFET model, indicating that while slower than simpler traditional electronic component models (i.e., less than level 3 MOSFETs), the model is sufficient for modest scale simulations and elimination of higher order terms that slow the simulation is prudent. Having shown time efficient and sufficiently accurate simulation of real memristive devices, the memristor model was then used for developing memristive hardware, eventually serving as the basis for conversion to a PyTorch model for large-scale (>500 input) neural network simulations.

Due to the enormous amount of computational resources required to simulate thousands of time-dependent and interconnected circuit elements on a hardware level, a PyTorch model was developed to simplify transient current–voltage relationships into flux-linkage timesteps. Since flux-linkage is the core internal variable for our FLCA dynamical memristors, the overall memristance change per device is approximated to be proportional to the total flux-linkage passed through the device for that timestep. This simplification can be made because the signal durations used in this work, during which the memristance would be physically transient, are short, typically 1 ms. Therefore, Eq. (1) is performed in discreet timesteps rather than being a continuous function. However, the total flux-linkage during training cycles is very significant, and characteristics such as magnitude dependence and voltage-dependent flux-linkage saturation must be accounted for. Thus, Eq. (4) is performed within the model by a rank 2 tensor in conjunction with the relevant conditional statements. Equation (2) only becomes relevant during predictions and is thus calculated after moving flux-linkage tensors from GPU memory back to memory that is accessible by the CPU. Separate tensors are used for input stimuli, flux-linkage for each dynamical memristor, as well as the tensor used to process Eq. (4). Both SPICE and PyTorch models are run on an inexpensive consumer-grade computer, highlighting compactness and efficiency.

## 3. Simulations of temporally dynamic neural circuits

**Figure 2:**
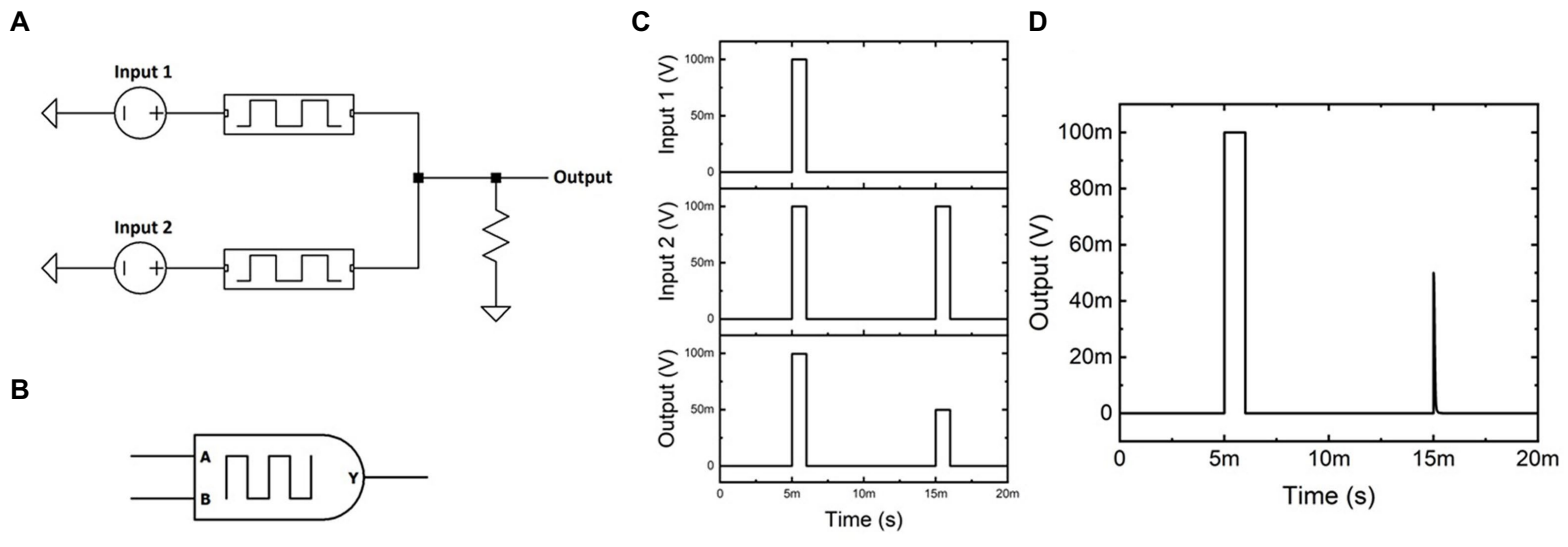
**(A)** Circuit schematic for VALVeD inputs using volatile memristors. **(B)** Circuit symbol for VALVeD inputs. **(C)** Transients of Input 1, Input 2, and the resulting Output when the volatile memristors are replaced with fixed resistors, resulting in a half-amplitude output for dissimilar inputs due to voltage division. **(D)** Transients of the Output using VALVeD inputs showing a heavily suppressed output for dissimilar inputs, thereby behaving in the same way as a logic AND gate.

The memristor model can thus be used to demonstrate memristive circuits that perform in-memory computation. Although many novel circuits were developed to showcase the usefulness of memristors in hardware, including the RCA and circuit logic that utilizes non-volatile passive memory (Supplementary Figures 4–7), this work will focus on time-dependent memristive circuits and their biologically inspired uses. Figure 2A shows the circuit schematic of the biasing architecture used to form Volatile AND Logic VoltagE Divided (VALVeD) computation. In this example, the two inputs are each serially connected to a volatile memristor ($A=50k,D=1\;ks^{-1},R_{0}=100\;\Omega \text{,}$ and $R_{3}=500\;kC^{-1}$) that then combine to form a current-summed output. The linked volatile memristors respond to the difference between their corresponding inputs, with the output voltage measured over a grounded resistive load. A circuit symbol for the VALVeD inputs (without the resistive load) is visualized in Figure 2B.

Using fixed resistors instead of volatile memristors results in the output transients in Figure 2C. When both inputs are identical, for example a digital HIGH (100 mV in this example), the resulting output is also identical to the inputs (digital HIGH). However, when Input 1 is digital LOW and Input 2 is digital HIGH, the dissimilar inputs result in a half-amplitude (50 mV) output due to voltage division over the fixed resistors. When volatile memristors are used, Figure 2D shows the output transients using VALVeD inputs and the same input signals as Figure 2C. Although the output for dissimilar inputs is initially half the input (50 mV) due to the instantaneous effect of the voltage divider, the volatile memristors quickly (tunable using parameter *D*) respond to the potential difference and suppress the rest of the pulse. Each input signal is a 1 ms rectangular pulse of 100 mV, or a flux-linkage of 100 μVs, which results in a 50 mV pulse of 1 ms duration at the output for the original implementation in Figure 2C with only resistors and only one active input. Thus, the resistive voltage divider is only able to reduce the input by 50% with a total flux-linkage of 50 μVs. In contrast, the output from the VALVeD inputs is heavily suppressed, with a total flux-linkage of 3.2 μVs calculated from the area under the output voltage trace of the 2^nd^ pulse in Figure 2D. Thus, when only one input is digital HIGH, the VALVeD inputs are able to suppress 96.8% of the original input, or effectively a digital LOW. The architecture functions as a leaky AND logic gate that allows similar analog signals pairs to pass while suppressing any other combination of inputs, essentially implementing a correlation function. The truth table demonstrating AND logic is shown below in Table 1. While the VALVeD inputs may be applicable as an AND gate for conventional digital circuitry, this work will demonstrate the use of VALVeD inputs in an analog, memristive neural network. When analog signals are used that are between the digital limits described here, the passed total flux-linkage is proportional to the correlation of the two signals.

**Table 1:** Effective truth table for the VALVeD input architecture with the behavior of a leaky AND logic gate for analog signals.

Input A	Input B	Output Y
OFF	OFF	0%
OFF	ON	3.2%
ON	OFF	3.2%
ON	ON	100%

**Figure 3:**
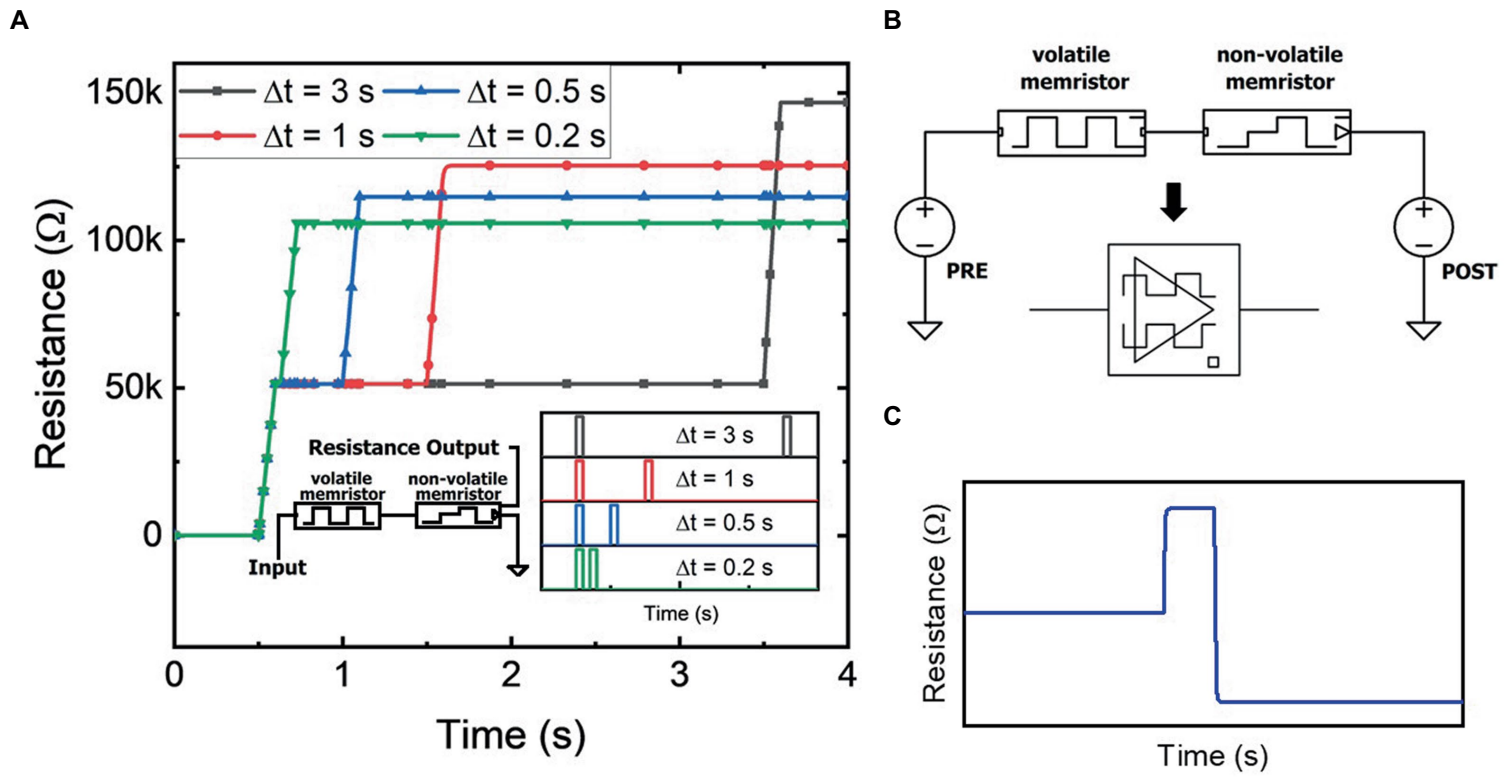
**(A)** Resistance of the non-volatile memristor in the desensitization circuit (left inset) from two identical input pulses with varied time separation (right inset). **(B)** Bidirectional memristor temporal circuit diagram and circuit symbol. **(C)** Resistance of the non-volatile memristor in the memristive circuit from bidirectional pulses within the temporal window. The overall decrease in resistance due to the temporal relationship between the input pulses demonstrates the effect of sensitization or paired-pulse facilitation.

The left side inset of Figure 3A shows the circuit schematic of a memristive circuit building block that consists of a volatile memristor in series with a non-volatile memristor (where for both the volatile and non-volatile devices, $A=1,D=1\;s^{-1},R_{0}=100\;\Omega \text{,}$ and $R_{1}=1\;MC^{-1})$. This is a physically realistic case where both devices can be made on the same Li_x_NbO_2_ chip with similar dimensions but different contact metals controlling (non-)volatility (Zivasatienraj et al., 2020). In this neuromorphic example, the volatile memristor enables desensitization of the non-volatile memristor to frequent stimuli. A stimulus source applies voltage pulses to the series-connected components and the resistance of the non-volatile memristor is measured as the output. The input stimulus consists of a pair of 1 V pulses, with a varied time separation between the two pulses, as shown in the right side inset of Figure 3A. Thus, the total input energy into the circuit is equal for each scenario. However, as shown in Figure 3A, the memristor’s final resistance differs for each temporal scenario as the initial pulse temporarily desensitizes the effect of subsequent pulses. Specifically, in response to the initial stimulus pulse, the volatile memristor is programmed to a higher resistance, which lowers the voltage and thus, total flux-linkage across the non-volatile memristor. The programmed resistance in the volatile memristor decays with time, thereby decreasing the desensitization effect for pulses with a larger time separation. If the time between each input pulse is sufficiently large, then desensitization does not occur. The temporal computation effect shown mimics the biological desensitization behavior seen in neurophysiology (Thesleff, 1959).

As shown in Figure 3B, the same memristive circuit can be stimulated by two voltage sources, a presynaptic and postsynaptic signal, rather than the single source shown previously. The compact circuit symbol for the bidirectional memristive circuit is shown below the circuit schematic in Figure 3B and will later be used as a building block for higher complexity circuits. In the bidirectional mode of operation, initial pulses to the volatile memristor enables sensitization of the non-volatile memristor to the stimulus pulses from the other side of the circuit. As shown in Figure 3C, the first pulse, $V_{PRE}$, temporarily programs the volatile memristor to a higher resistance. A response pulse, $V_{\mathrm{POST}}$, is then sourced from the other side of the circuit while being within the temporal window of the volatile memristor. Effectively, $V_{PRE}$ and $V_{\mathrm{POST}}$, while of identical polarity, because of their locations in the circuit act on the memristive circuit as if they are of opposite polarity. Thus, the volatile memristor reacts by transitioning from a high resistance state, through its initial low resistance state, and then back to a higher resistance state. This behavior is consistent with memristive volatile operation that results in the pinched hysteretic current–voltage bow-tie curve (Chua, 2014). Due to the transition between resistance states, the overall transient resistance is reduced. As shown in Figure 3C, the non-volatile memristor thus receives a higher flux-linkage and is programmed to a resistance state lower than its initial state prior to $V_{PRE}$. The reverse can also be shown: a $V_{\mathrm{POST}}$ before $V_{PRE}$ pulse results in a net increase in the resistance of the non-volatile memristor. The dynamical memristive circuit thus demonstrates an enhanced response to consecutive pulses, mimicking synaptic facilitation phenomena such as PPF as seen in neurophysiology (López, 2001).

**Figure 4:**
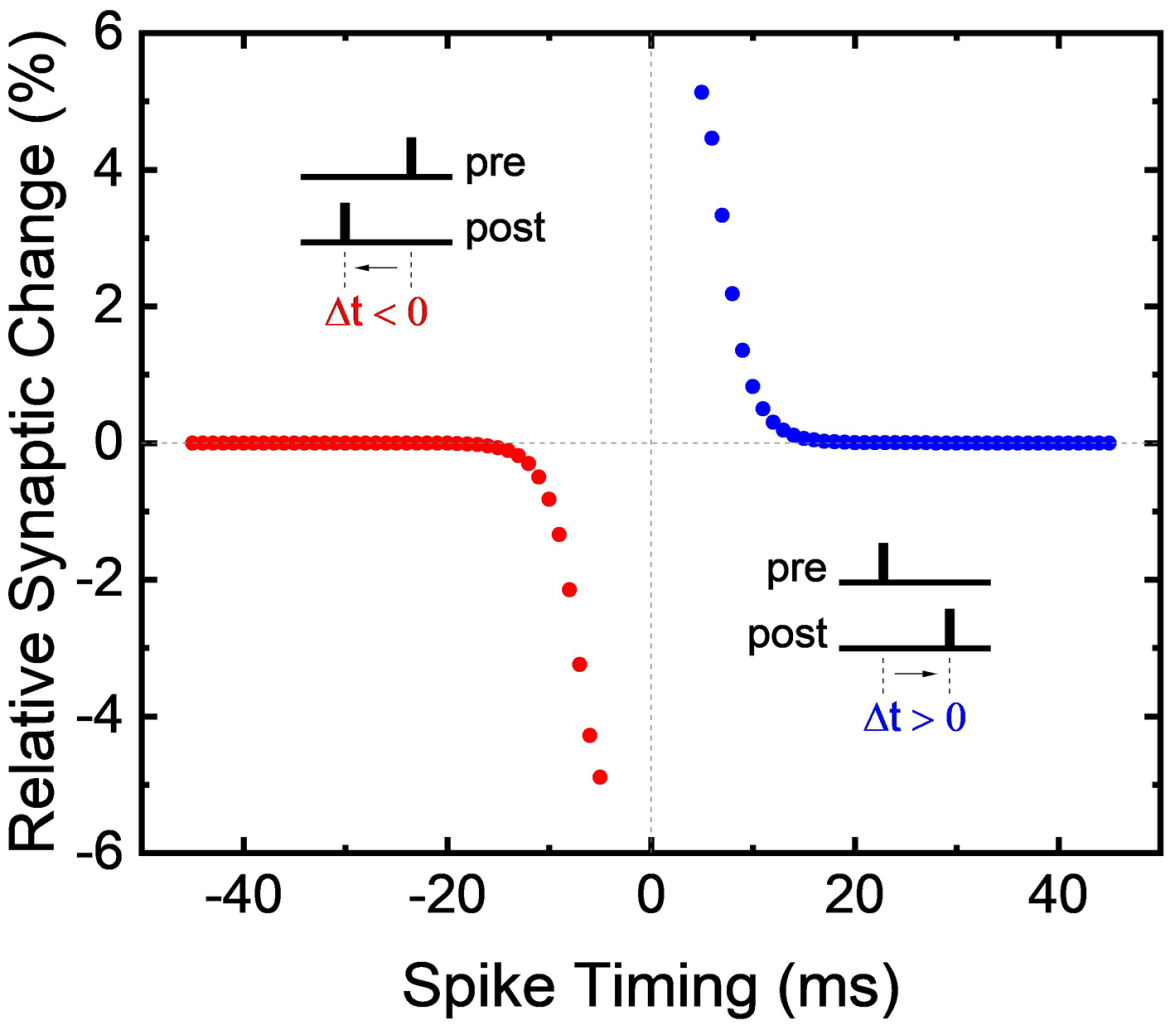
Spike-timing-dependent plasticity demonstrated using a bidirectional memristive circuit.

Utilizing the synaptic facilitation exhibited by the bidirectional memristive circuit in Figure 3B, the biological phenomenon of STDP is replicated and the renown anti-bell curve is shown in Figure 4. If $V_{PRE}$ precedes $V_{\mathrm{POST}}$, the conductivity of the non-volatile memristor increases to strengthen the synaptic connection. Conversely, if $V_{\mathrm{POST}}$ precedes $V_{PRE}$, the synaptic connection is weakened by an increase of resistance in the non-volatile memristor. The magnitude of change in synaptic weight is dictated by the time between the pair of pulses. Shorter delays result in larger changes to the synaptic connectivity, while pulse pairs that fall outside the temporal window do not alter the synaptic weight. Importantly, this demonstration of STDP does not require pulses to overlap, nor is proper functionality contingent on the shape of each action potential. Generic rectangular voltage pulses are used for all voltage stimuli described in this work, but owing to its origins in flux-linkage, bio-realistic or even heterogeneous neural signals could be used as well as magnitude, frequency, phase, or pulse shape encoding. In addition, the memristive circuits mimic biological processes such as desensitization, PPF, and STDP without incorporating external elements such as capacitors or microprocessors. While the dynamical memristive circuits are diversely applicable to a broad range of leaning methods, STDP is demonstrated here given its widely understood function.

## 4. STDP-enabled physical neural networks with lifelong self-learning

STDP is a form of Hebbian learning that determines the weight of synaptic connections between neurons. When adopted as a learning algorithm in a spiking neural network (SNN), STDP provides a self-learning process that enables computational weights within the SNN to be individually self-updated *via* the temporal relationship between spikes exchanged by neurons. This is especially meaningful for physical neural networks because manually addressing and manipulating individual weights, such as in the process of BP, is an energy intensive process that also increases system latency and size. Thus, SNNs are garnering heavy interest as a low power and biomimetic neuromorphic solution (Kim et al., 2021). Here, dynamical memristors are used to build an STDP-enabled self-training physical SNN.

**Figure 5:**
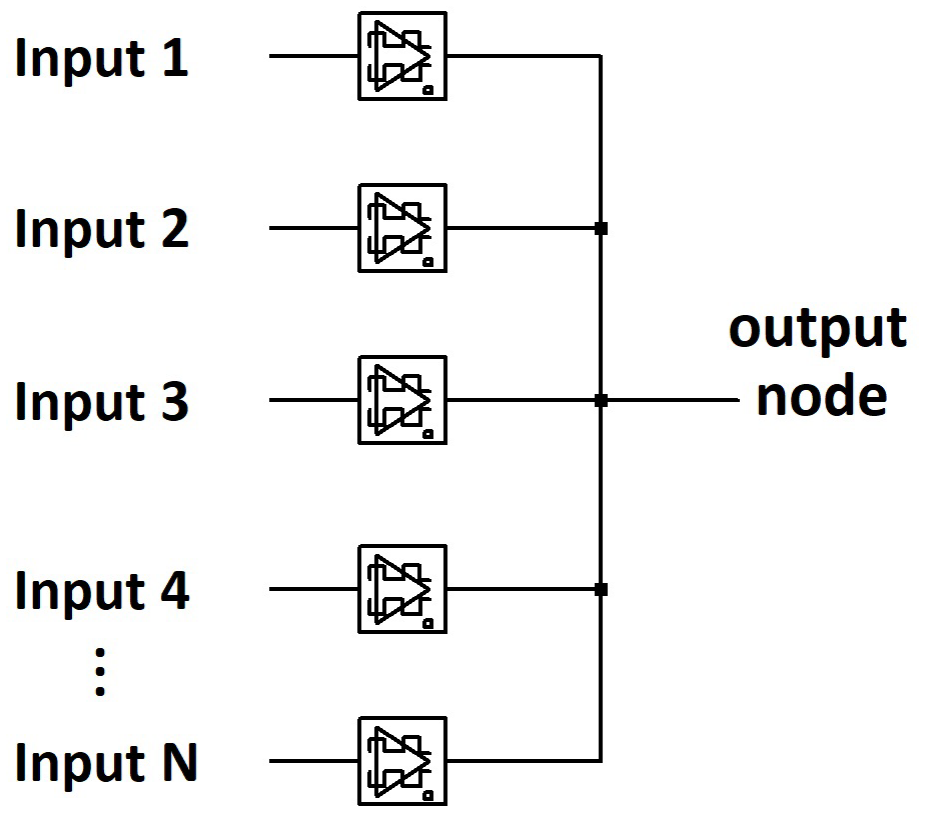
Abbreviated circuit schematic for a single layer memristive physical network capable of self-training *via* STDP.

Figure 5 shows an abbreviated circuit schematic for a single layer memristive physical neural network. For N number of inputs, there are N dynamical memristor components that accept one input each before being pooled into an output node. Albeit a demonstration, this architecture is significantly smaller than typical neural network layers consisting of interconnected nodes. Each dynamical memristor in the network is comprised of one non-volatile and one volatile memristor, as shown previously in Figure 3B. Thus, no signal has direct connection to merely one electronic component, as is the case with RCA alternatives. The weight of a connection within the memristive neural network is determined by the conductivity of the non-volatile memristor, while the volatile memristor performs temporally dynamic calculations for learning. In the example of an image classifying perceptron, each input pixel, represented in hardware by a voltage source, is wired directly to a dynamical memristor circuit. All the memristive weights are then connected to a shared output node to result in a current-summed output for each image. For the 28×28-pixel images of handwritten numerical digits from the EMNIST dataset (Cohen et al., 2017), the 784 pixels from each grayscale image is serialized into 784 voltage sources as inputs into the memristive neural network. Every input image translates to a 1 ms duration voltage pulse from all the inputs simultaneously. The input amplitude from each voltage source is scaled to the intensity of the represented pixel normalized between 0 V and 100 mV, with most inputs per image being zero as is the nature of written characters (there are more ‘inactive’ pixels than there are pixels ‘actively’ forming the digit/letter). Every input image is then reciprocated by a system-wide training pulse that is timed within the temporal window for STDP. Thus, rather than BP, the network is trained by a singular 100 mV voltage signal at the output node that propagates throughout the entire SNN, bio-realistically implementing a multi (768) stimuli, single response neural network (see Supplementary Figure 7). Each memristive perceptron is trained on a numerical digit from the EMNIST dataset. As is the case for all stimuli within this network, every pulse is a 1 ms duration rectangular waveform. During the training phase, the timing of the training signal is reliant on whether the targeted digit is indicated in the labeled dataset. Using STDP, the memristive weights are self-trained to recognize the handwritten digits.

**Figure 6:**
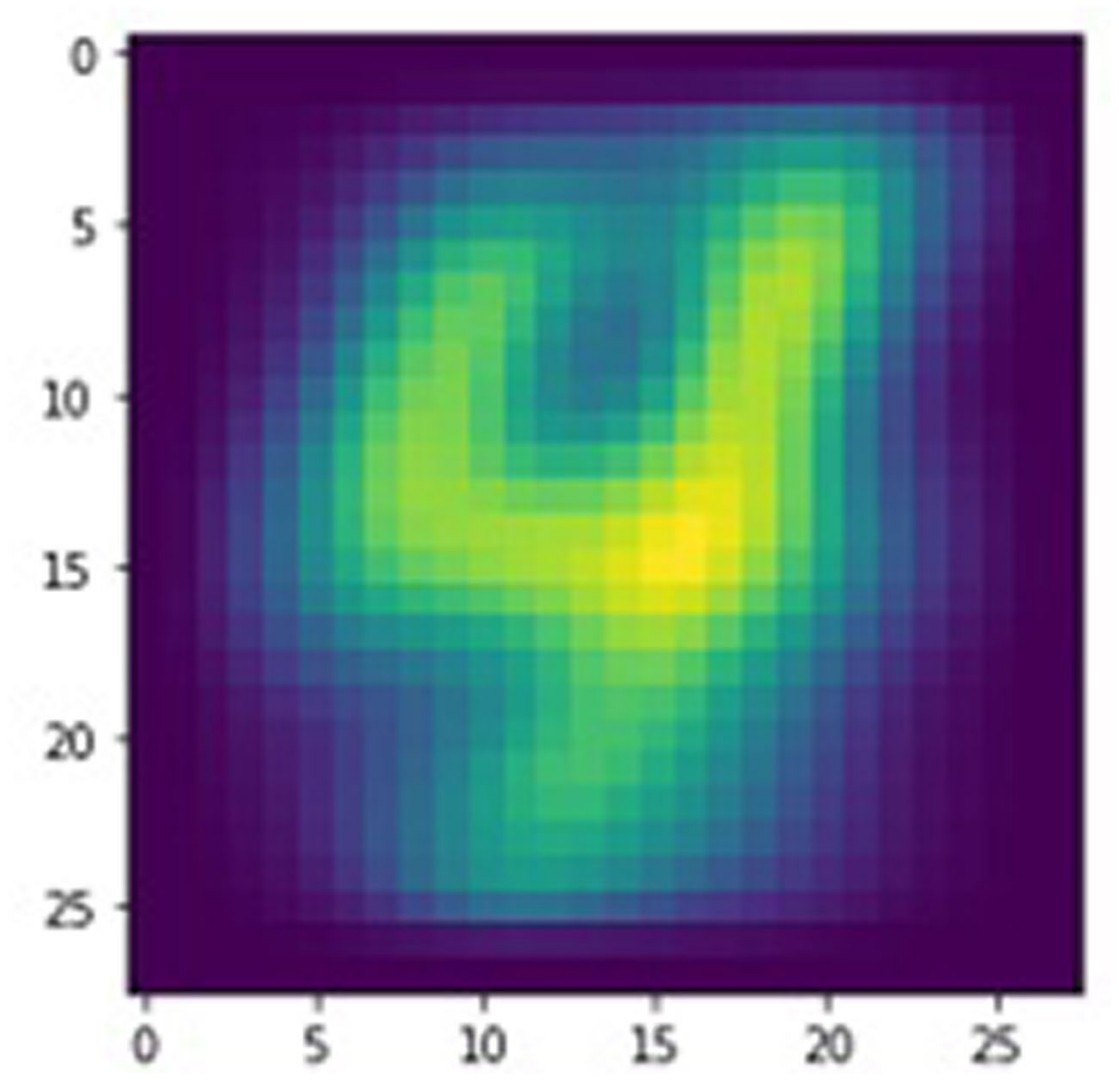
Visualization of physical neural network trained on a handwritten numerical digit.

As an example, the digit “4” was self-trained on the dynamical memristive neural network. The timing of the response pulse for each training image thus depends on whether the labeled training data indicates that the target, the numerical digit “4,” is pictured in the input image. The time delay between the input and response stimuli is 250 ms for images that do not contain the target. In contrast, a 10 ms delay is used for training images that picture the digit “4,” thereby ensuring that the response pulse falls within the temporal window for STDP, as previously shown in Figure 4. This pairwise learning protocol is repeated for only a quarter of an epoch, or 60,000 of the 240,000 training images from the randomized EMNIST dataset before convergence was achieved, indicating successful self-training of the SNN. As shown in Figure 6, the state of the neural network is visualized by arranging the 784 memristive weights back into a 28×28-pixel array with normalized pixel intensities tied to the conductivity of each non-volatile memristor. The distinct shape of the numerical digit “4” is observed, indicating that the physical neural network was successfully trained. A conservative estimate of the energy required to train the network is calculated to be 1.6 μJ per training image by assuming the lowest resistance (and thus highest current) for each memristive weight in the network. Although more expensive per training image than some advanced training algorithms that have an energy consumption of 71.3 nJ per image (Choi et al., 2022), the more compact network and quicker learning rate of the dynamical memristive neural network results in an ultralow total training energy of 94 mJ before convergence. Conventional neural networks that use backpropagation require hundreds of epochs to train a network towards convergence, resulting in a total energy consumption of 1.7–6.4 J depending on the emphasis placed on energy efficiency. However, inference operations still cost between 53 nJ and 1 μJ for these large neural networks of >198 k weights. The dynamical memristive neural network is able to make predictions using only 78 pJ per inference due to the use of analog MAC operations performed simultaneously. Using a simplified classification scheme as an example, input images containing the target digit will experience an overall lower resistance to the output node, allowing for binary classification of the target digit. In contrast, other input digits are forced to utilize untrained and thus more resistive paths to the output. By using a sufficiently small or fast (beyond the ion response bandwidth; Ghosh et al., 2023) test signal or AC test signals balanced in positive and negative flux-linkage, the overall network resistance can be sampled without modifying memristances while consuming ultralow energy. Adopting STDP as a learning rule, the memristive weights were able to efficiently self-train towards a desired outcome without manually addressing any of the individual weights, instead relying on the temporal relationship between the input pulses and the system-wide responses.

**Figure 7:**
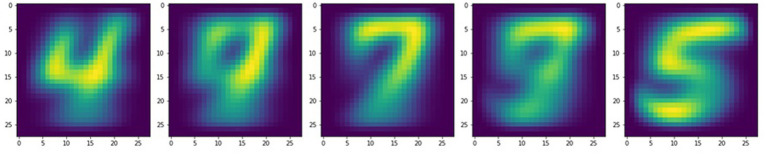
Visualizations of the memristive neural network demonstrating lifelong self-learning through successful training on different numerical digits consecutively.

In addition, the self-learning properties of this physical SNN can be used to implement lifelong learning. Typically, a neural network is trained to perform a task and must be expensively retrained to accommodate any changes, correct errors, or counter drifting parameters. In contrast, the dynamical memristive network continuously learns even as computations are being performed, provided that large enough input and training signals are used to program the dynamical memristors (small signals result in only predictions at the output without any training, much like typical neural networks). Thus, the target digit for training can be altered or completely changed and the memristive layer is able to quickly readjust. Using the visualization technique mentioned earlier, Figure 7 shows the evolution of the memristive network as the training target is changed multiple times within the same training instance. For consecutive training targets, a video is formed of handwritten digits gradually morphing into other numbers as the dynamical memristive network readjusts itself on demand. The video can be viewed from the Supplementary Materials.

This implementation of the STDP-enabled physical neural network is only capable of learning one target (or one alphanumerical character) at a time. To resolve this limitation, multiple memristive layers were trained in parallel, each with different target digits from the EMNIST numerical dataset. The output from each memristive network layer is compared using a winner-take-all strategy that highlights the speed of training, resulting in >70% prediction accuracy after training for as little as ¼ an epoch. Since the focus of our work is on self-learning in hardware using newly envisioned architecture, we did not implement more complex classification approaches. The simplistic winner-take-all classification mechanism handicaps the present accuracy and future implementations will utilize more complex classification approaches. However, such simplified classification methods do not incorporate the benefits of the analog infrastructure of the physical network. Since the dynamical memristive SNN was designed with capability for bidirectional operation, the current-summed output may be embedded inside other layers. For example, the response signal can be the output from another memristive layer, eventually establishing a cohesive self-learning system. The use of system-wide and generic rectangular pulses provides flexibility in interlayer communication, and the temporal dynamics necessary for STDP can be achieved using volatile memristors or by encoding the transmission line delays that result from signals passing through layers of memristive hardware. Complex training approaches can also enhance the dynamical memristive neural network, such as the use of irregularly timed inputs that utilize the full temporal window of STDP. Although these endeavors are beyond the scope of this research, this work validates the foundation for using analog memristors as computing elements in a self-training neural network that is capable of self-learning without direct access to the individual weights.

## 5. Self-training architectures *via* introspective associative learning

**Figure 8:**
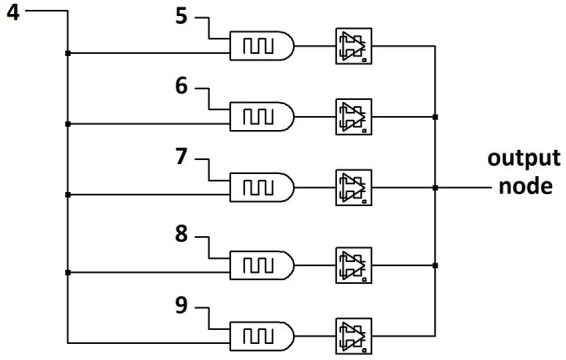
Partial circuit schematic for the PILL architecture. Inputs are paired together to become VALVeD inputs for the memristive weights, which are then pooled into an output node.

Figure 8 shows the partial circuit schematic for the Paired Input Learning Layer (PILL) architecture which can perform a similar function as the STDP approach above but absent the use of STDP. Each input into the network, which represents a pixel of an input image in the exemplary task of image classification, is paired with every other input without duplicates, implementing the mathematical combination function, $C_{i}^{k}$, with i=2 and k equal to the number of pixels in the input image. The current of each input pair is summed using the VALVeD input circuit which feeds into a dynamical memristor before summing to the read output node. Figure 8 only shows a subset of the paired inputs, but the full layer would have all inputs paired with all other inputs and without any duplicate pairs, resulting in $C_{2}^{9}=36$ paired inputs in the simplified case of a 3 × 3 image. The pairing algorithm shown in Figure 8 does not show pairing of input 4 with any input numerically less than 4 to eliminate duplication of pairs. Importantly, a fully connected network has one or more internal hidden layer typically with more nodes than the input layer in order to increase prediction accuracy. The PILL approach dramatically lowers the number of memristors needed to implement the network, though larger than the previous example due to the additional functionality to be discussed shortly. For example, if a minimal size fully connected network with only one hidden internal layer and one output node has m hidden layer nodes and there are k inputs, the minimal number of memristor synaptic weights is $m\left(k+1\right)$. Because the PILL architecture scales as $C_{i}^{k}$, the PILL network size is dramatically smaller than a typical fully connected network. For all examples studied here, only one PILL resides between the input layer and the output layer, although more PILLs would potentially enhance learning capability at the cost of computational complexity.

The PILL architecture can be operated using STDP with the same training protocol as described previously, where each input image receives a system-wide response pulse that is timed within the temporal window for STDP. To classify the 28×28-pixel images of handwritten numerical digits from the EMNIST dataset, the PILL would consist of only $C_{2}^{784}=306,936$ sets of VALVeD inputs and memristive weights. STDP-based supervised training of the network results in visualized outputs of self-trained weights similar to that of the physical SNN shown previously in Figures 5, 6. Although the training and prediction results are comparable, the learning mechanism is different because of the more complex PILL architecture. Rather than learning the association between the individual input pixels and the desired pixel intensity, the input-paired network in the PILL architecture allows the dynamical memristors to associate pairs of input pixels to the overall target image, essentially implementing an inverse derivative function that highlights correlations in two pixels. Both inputs of the pair affect the training signal to the dynamical memristor due to the prior current summation, ultimately resulting in self-learned associations amongst memristor pairs within the same network layer. The introspective associative learning enables the PILL network to be trained on a limited subset of multiple targets simultaneously. In addition, this computational feature originates from the PILL architecture and is not reliant on STDP.

**Figure 9:**
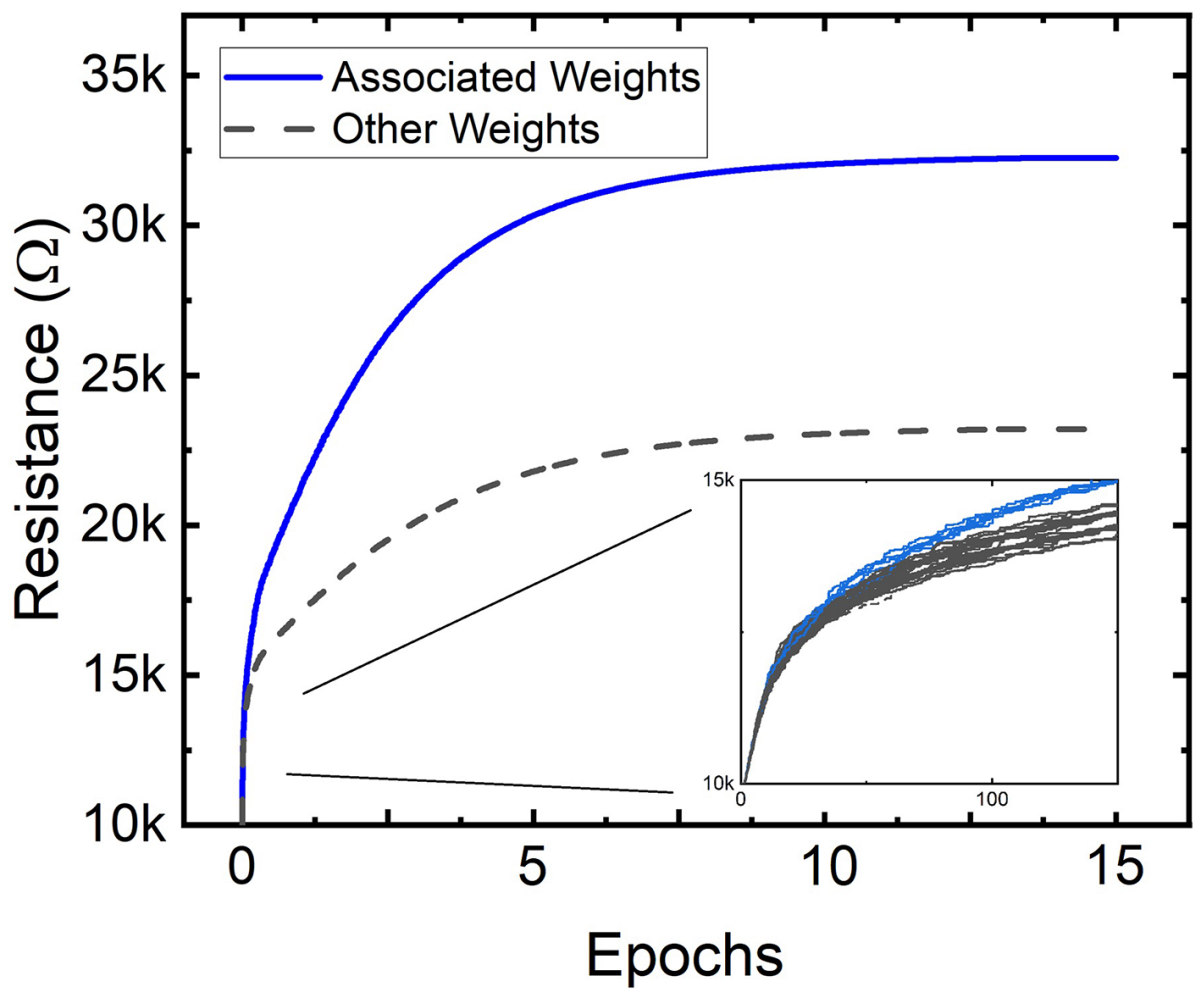
Resistances of all the non-volatile memristors within the memristive PILL architecture being trained *via* multiple epochs. The separation of resistance states is indicative of successful training. Since there are only two groups of weights (resistances), two colors are used to represent two resistance values for associated and non-associated weights.

To demonstrate the pixel-to-pixel introspective associative self-learning, a simplified task of classifying vertical lines anywhere within a 3×3-pixel image is described. The single-layer PILL architecture thus consists of $C_{2}^{9}=36$ VALVeD input pairs and memristive weights. To demonstrate alternative learning mechanisms, this example uses the mere presence of a system-wide response to train the SNN rather than STDP. The system-wide training signal is pulsed alongside input images that contain a vertical line. Input images that do not contain a 2-pixel or 3-pixel vertical line anywhere within the image do not receive a response pulse. Figure 9 shows the programmed resistance transients of all the dynamical memristors in the PILL during training. All combinations of inputs are represented in each epoch and in a randomized sequence. The training signal amplifies the memristor programming for input images containing vertical lines. The clearly divergent resistance states shown in Figure 9 indicates that the training process has produced unequal changes within the dynamical memristive layer. The weights associated with the inputs forming vertical lines anywhere within the image were trained to higher resistance states than the weights that were not associated with vertical lines. To clarify, a subset of the memristive weights is driven by paired inputs for pixels that are associated with the formation of a 2-pixel or 3-pixel vertical line in the input image. For example, in a 3×3 formation the pixels 1, 4, and 7 form a vertical line in the left-most column, and thus the VALVeD pairing of these pixels (1–4, 4–7, and 1–7) are represented as memristors that associate to that vertical line. These memristive weights, when an input image containing a line is processed by the PILL, experience amplified programming due to the training signal and are thus labeled as “associated weights” in Figure 9. Most of the weights in the layer are from input pairs that are not associated with the formation of a vertical line and are statistically suppressed from enhanced programming, thus resulting in a lower resistance state. This divergent memristance result was repeatedly confirmed using both 2 and 3-pixel lines, and with all permutations of singular and multiple vertical lines within the image.

Importantly, the PILL architecture was trained using generic system-wide pulses that were delivered through the output node whenever the input image contained a vertical line anywhere within the image and without the use of convolution. Since pixel-to-pixel associations are learned rather than the association between individual pixels to desired values, the training on a specific location of the vertical line within the 3×3-pixel image does not statistically overwrite any prior training of the network. Thus, all locations of the vertical line are trained simultaneously and within the same epoch. Although not required for successful operation, the VALVeD inputs bolster this learning mechanism by suppressing unintentional programming of dynamical memristors for input pairs that are unalike. In this simplified example, all weights associated with permutations of 2 or 3-pixel vertical lines were trained to a higher resistance concurrently. Thus, the trained PILL can now perform useful computations, such as responding differently to images with and without lines anywhere within the image, without needing to convolve through a subset of the input image to recognize the pattern. Although this approach undoubtedly has limitations on the types of variation allowed between the multiple simultaneous training targets, the introspective associative learning allows for unique analog computations to be performed on the entire input vector in hardware without using expensive algorithms such as convolution or BP. Additionally, self-learning in the PILL architecture is robust to noisy or corrupt data due to statistical averaging of the associated and unassociated memristive weight training.

## 6. Concluding remarks

A physical solution for non-von Neumann computation is discussed that exploits the in-memory computation and temporal versatility of intercalation-based memristors. A compact phenomenological memristor model is presented, including several tunable parameters that enable accurate emulation of non-linear and asymmetric behaviors found in experimental devices. Dynamical memristors were then simulated in neuromorphic circuitry to mimic biological processes, including desensitization, sensitization, PPF, and STDP. To demonstrate computational examples using dynamical memristive hardware, a self-training neural network was shown that uses dynamical memristors and STDP as a learning rule. The use of a neuromorphic learning rule such as STDP allowed the neural weights to self-update according to the temporal relationship between inputs and system-wide responses rather than training through the expensive process of backpropagation and individual memristor manipulation. The dynamical memristive SNN was used as an image-classifying perceptron trained on the EMNIST dataset to show lifelong self-learning, showing no difficulty in continuously relearning different handwritten characters in succession. In addition, the PILL architecture was introduced, strengthened by VALVeD input architectures, to demonstrate introspective associative self-learning capable of classifying features in various locations of an image without convolution. Importantly, owing to the time integration implemented inside the dynamical memristor, these memristive architectures execute temporal computations using generic signal agnosticism. While simple rectangular pulses were used for demonstrations, heterogeneous and biologically relevant signals are compatible with the memristive computing infrastructure and could bring yet another level of computational ability. The examples in this work guide the design of neuromorphic hardware and validates the potential of using the in-memory computation ability featured in temporally dynamic memristors to perform complex functions for neuromorphic systems.

## Data availability statement

The raw data supporting the conclusions of this article will be made available by the authors, without undue reservation.

## Author contributions

All authors listed have made a substantial, direct, and intellectual contribution to the work and approved it for publication.

## Funding

This material is based on the work supported by the Air Force Office of Scientific Research (AFOSR) under Award No. FA9550-18-1-0024 managed by Ali Sayir.

## Conflict of interest

The authors declare that the research was conducted in the absence of any commercial or financial relationships that could be construed as a potential conflict of interest.

## Publisher’s note

All claims expressed in this article are solely those of the authors and do not necessarily represent those of their affiliated organizations, or those of the publisher, the editors and the reviewers. Any product that may be evaluated in this article, or claim that may be made by its manufacturer, is not guaranteed or endorsed by the publisher.
