# Supplementary material for: Dynamical memristive neural networks and associative self-learning architectures using biomimetic devices
Source: Front Neurosci. 2023 Apr 20;17:1153183. doi: 10.3389/fnins.2023.1153183 (PMC10157062; doi:10.3389/fnins.2023.1153183)
Supplement: Supplementary file 2 [file Data_Sheet_1.DOCX]

Supplementary Material

Chronomemristive Neural Networks and Associative Self-Learning Architectures using Biomimetic Devices

Bill Zivasatienraj and W. Alan Doolittle*

*** Correspondence:** W. Alan Doolittle: alan.doolittle@ece.gatech.edu

# Memristor Implementation in SPICE

The mathematical memristor model is implemented in SPICE using behavioral voltage sources and a behavioral resistor. Behavioral elements in SPICE allow for compact and efficient execution of mathematical functions and result in a ground-referenced voltage output that can be easily referenced elsewhere within the circuit.


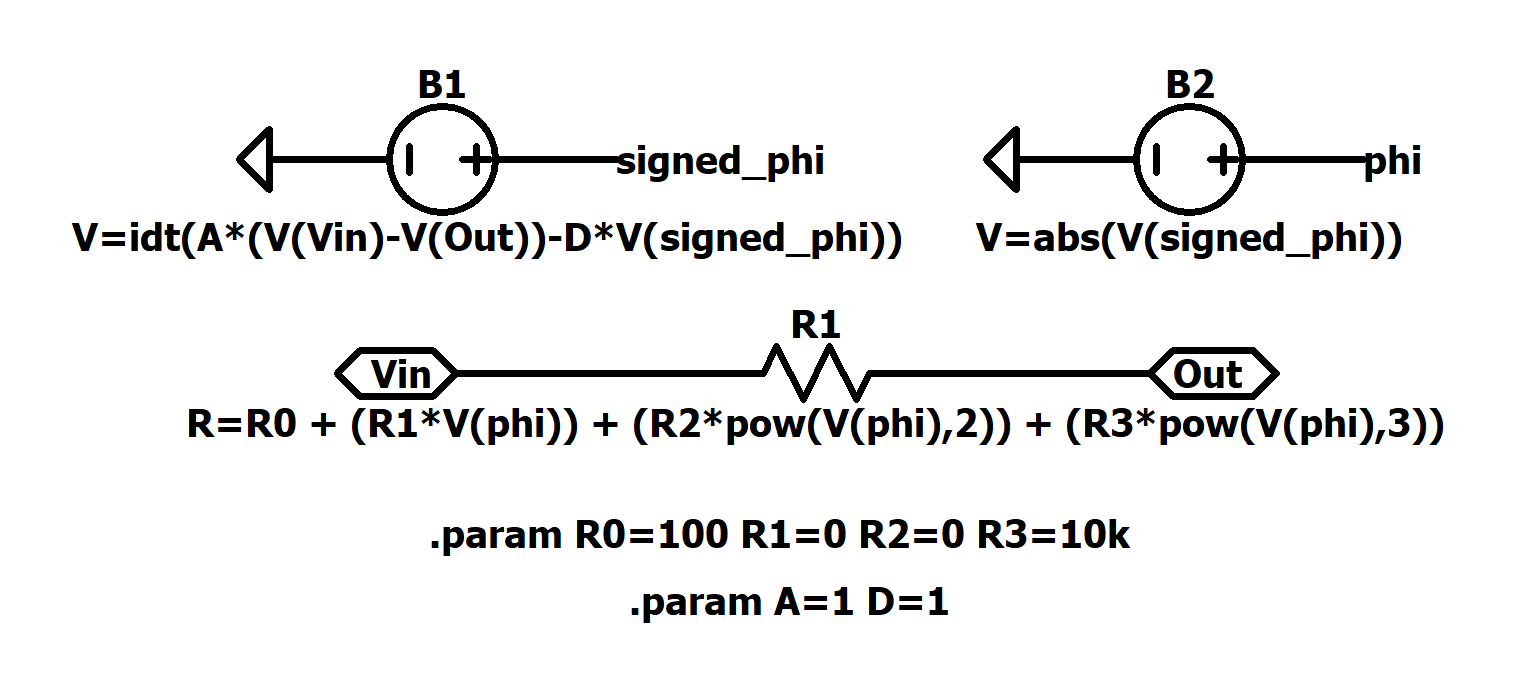


Supplementary Figure 1. Implementation of a volatile memristor in SPICE.

As shown in Supplementary Figure 1 for a volatile memristor, equation (3) is implemented in a behavioral voltage source to compute flux-linkage. All computational variables are parametrized in SPICE. Another behavioral voltage source is used to calculate the absolute value of the flux-linkage since our experimental memristors show bipolar operation in which resistance increases from the initial state regardless of applied polarity. Equation (2) is implemented in a behavioral resistor. The behavioral resistor is directly connected to the input and output terminals of the circuit element, thereby allowing SPICE to execute equation (1) pertaining to Kirchhoff’s circuit laws.


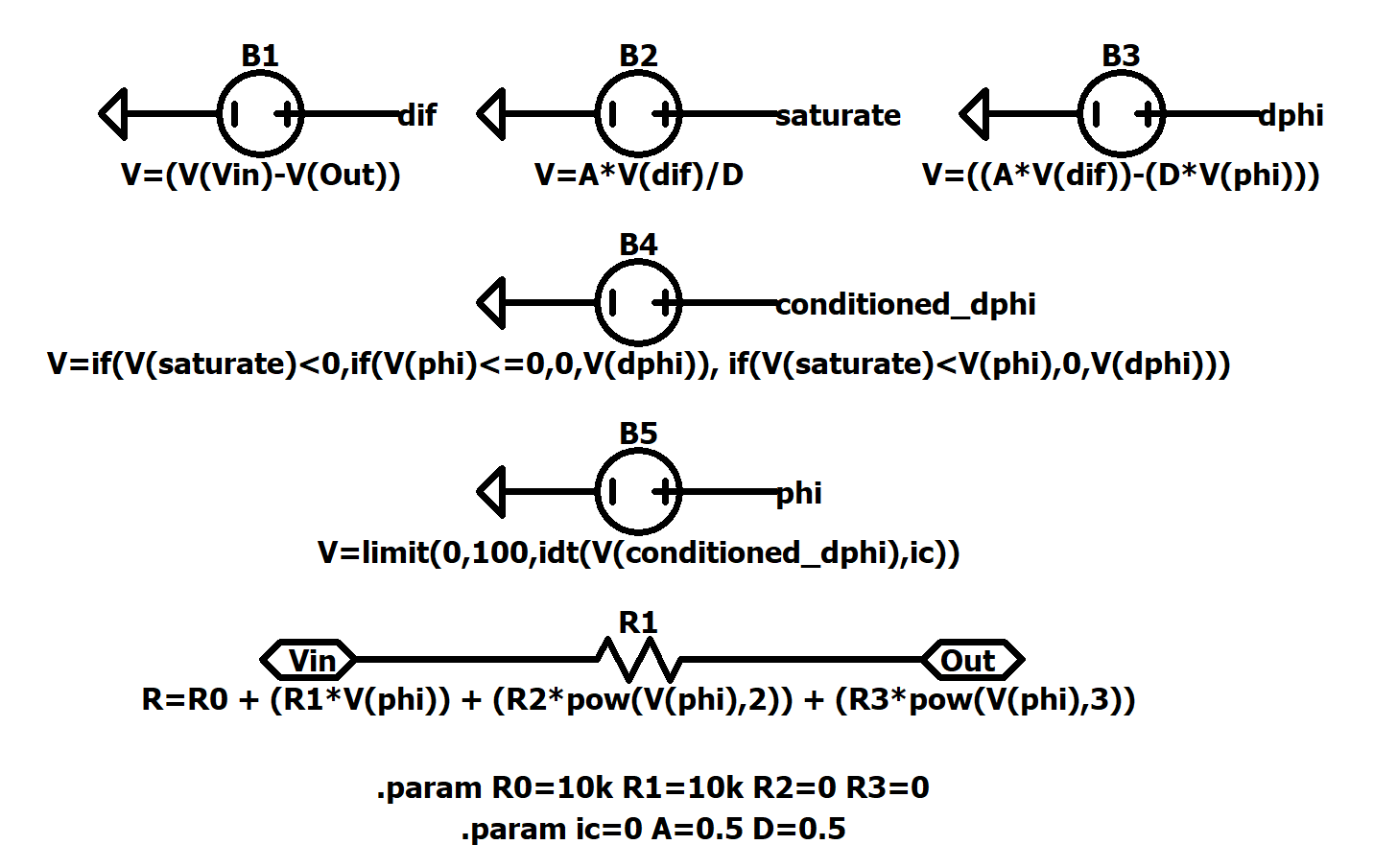


Supplementary Figure 2. Implementation of a non-volatile memristor in SPICE.

Similarly, Supplementary Figure 2 shows the SPICE implementation equations (1), (2), and (4) for a non-volatile memristor. More behavioral voltage sources are used to implement the conditional statements of equation (4). Optionally, as shown in behavioral source B5, the effective flux-linkage can be bounded arbitrarily. All non-volatile memristors in this work are bounded as shown in Supplementary Figure 2, which effectively results in only non-negative values for flux-linkage.

Supplementary Figure 3. The non-volatile memristor model tuned to show analog resistance changes that match experimental FLCA Li_x_NbO_2_ devices being programmed by consecutive 1 V pulses.

Supplementary Figure 3 shows how equations (1), (2), and (4) can be tuned to match the transient analog resistance of a previously published experimental Li_x_NbO_2_ non-volatile device programmed by consecutive 1 V pulses. The fitting parameters can be tuned within the SPICE model or separately using pragmatic scripts such as Python or MATLAB. In this case, parameter extraction on the experimental device data yielded $A=0.1, D=0.07 s^{-1}, R_{0}=167.6 \Omega,$ and $R_{1}=189 C^{-1}$.

# Simulations of Memristive Circuits

Memristors can perform computations in memory, thereby reducing the number of conventional transistors typically required to perform similar functions while featuring lower power consumption. The memristor model can thus serve as a guide for developing memristive circuits.


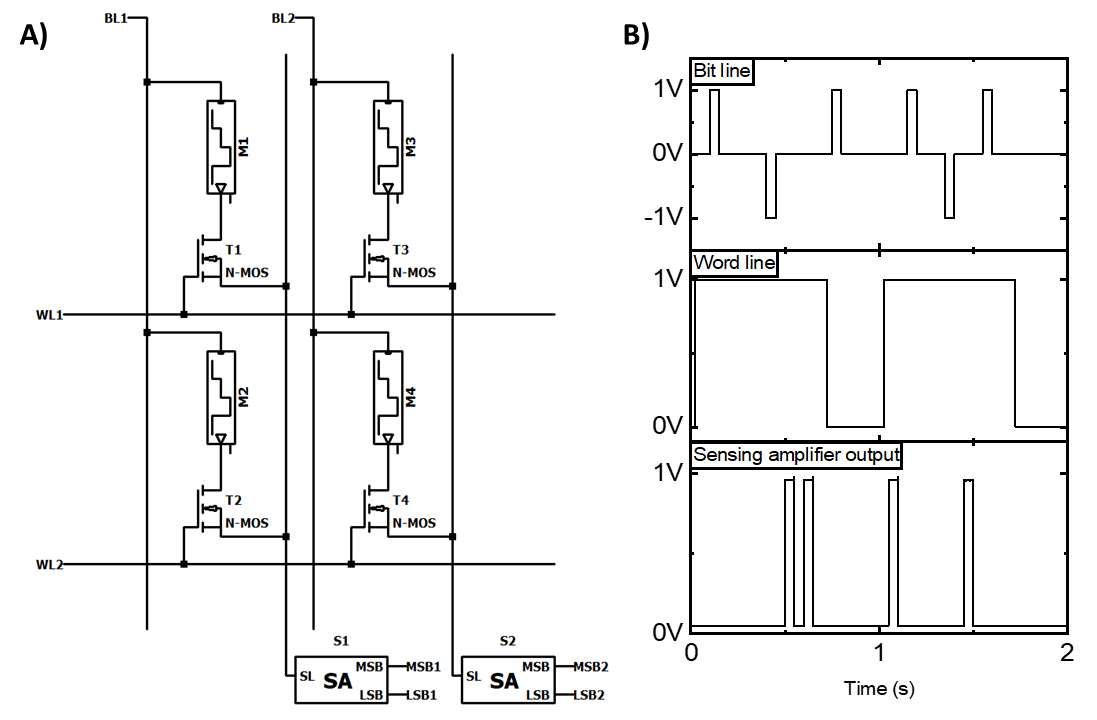


Supplementary Figure 4. A) Circuit schematic of a 2x2 1T1R array using one non-volatile memristor (M1-4) and one N-channel MOSFET (T1-4) per cell. The output is read using a sense amplifier (S1-2) on each sense line. B) Transient waveforms of a digital memory cell in multiple SET and RESET operations. The memory cell shows proper memory functionality from the sensing amplifier output.

To verify that the compact SPICE implementation of the memristor model is adept at simulating conventional memristive circuits, a 2x2 resistive random-access memory (RRAM) array is constructed in a one-transistor-one-resistor (1T1R) configuration, as shown in Supplementary Figure 4A. Supplementary Figure 4B shows the transient waveforms of the memory cell when the non-volatile memristor is tuned for binary switching operation ($A=1k, D=1k s^{-1}, R_{0}=1 k\Omega,$and $R_{1}=100 kC^{-1}$), much like a filamentary or phase change memristor. The memristive 1T1R cell shows proper memory functionality in multiple SET and RESET operations, with and without the word line enabled.


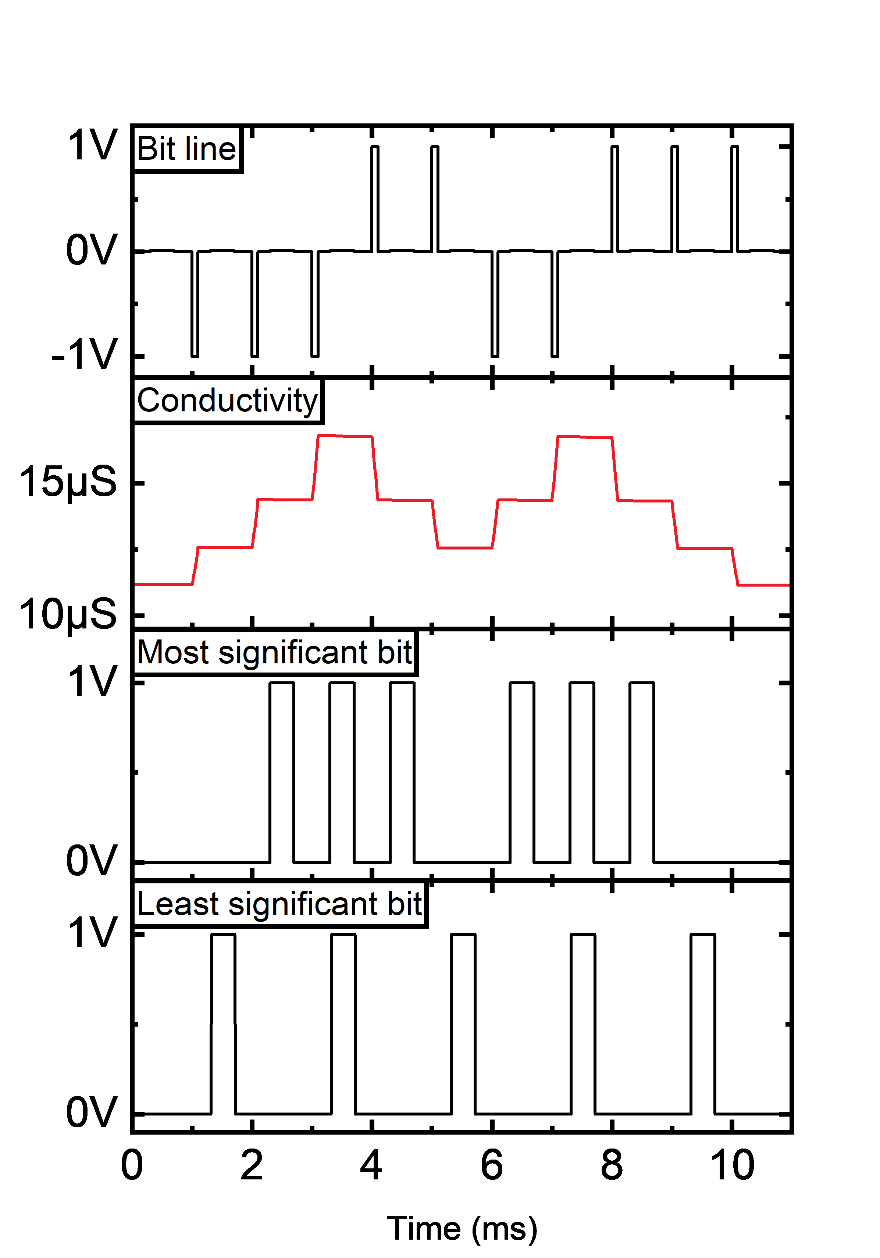


Supplementary Figure 5. Transient waveforms of a multi-level memory cell with the word line enabled. The non-volatile memristor is programmed to 4 distinct states that yield a 2-bit output from the sensing amplifier.

To emphasize the versatility of the flux-controlled memristor model, an RRAM array of identical structure is built, but the non-volatile memristors are set to analog, multi-level operation ($A=1k, D=1.5 s^{-1}, R_{0}=20 k\Omega,$and $R_{1}=10 kC^{-1}$, corresponding to a conservative subset resistance range, $60 k\Omega$ to $90 k\Omega$, of the experimentally verified Li_x_NbO_2_ memristor range, ~$2 k\Omega$ to $180 k\Omega$). Four distinct resistive states (2 bits) result upon programming for this example but in practice higher divisions of resistance could likewise be achieved. Supplementary Figure 5 shows the transient waveforms of the multi-level cell with the word line enabled, demonstrating 2-bit output from the sensing amplifier for each memory cell, totaling an 8-bit output from two sensing amplifiers for the 2x2 array. The flexibility of the FLCA memristor model allows simulations of both digital and analog memristors in an RRAM application simply by innate parameter manipulation.


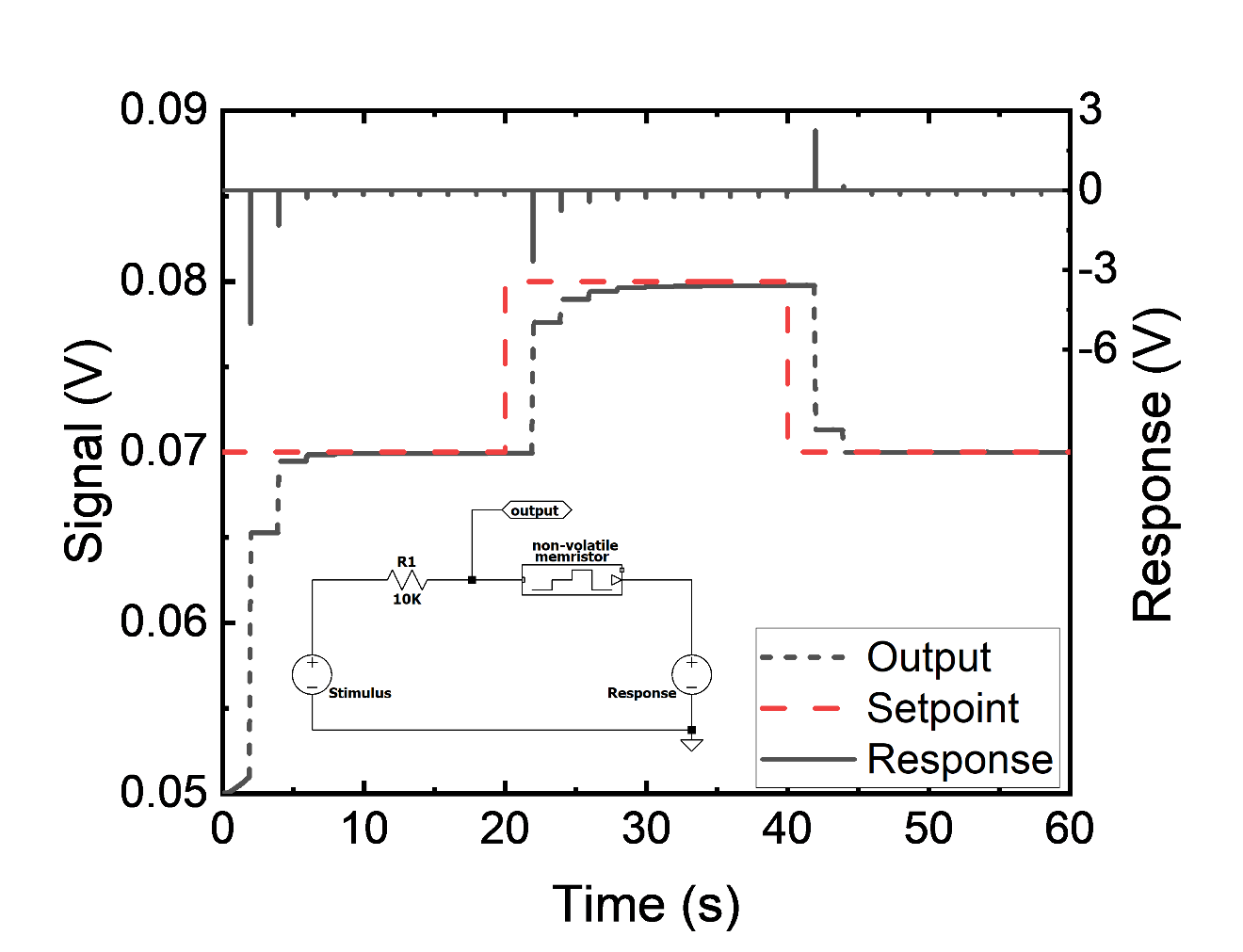


Supplementary Figure 6. The output of the setpoint stabilization circuit being trained to match a setpoint value. The response signal is proportional to the error between the output and setpoint values. The inset shows the schematic of the circuit.

The SPICE memristor model also enables the simulation of memristors in circuitry for biomimetic applications. The inset of Supplementary Figure 6 shows a biomimetic “stimulus/response” circuit implementing setpoint stabilization by using a constant-amplitude square wave to implement a non-varied stimulus that is sent through a fixed resistor in series with an emulated non-volatile memristor ($A=1, D=1 s^{-1}, R_{0}=15 k\Omega,$and $R_{1}=50 kC^{-1}$), forming a voltage divider for the output between the two resistive components. The setpoint values are chosen externally. The difference of the setpoint and the circuit’s output is the error, which is also computed externally. A response signal proportional to the error is then used to train the non-volatile memristor to resistance values that would shift the voltage divider output towards the setpoint signal and correct the error. Supplementary Figure 6 shows the output of the voltage divider, with training pulses filtered out via sampling, being calibrated to meet various setpoint values by training the memristor. The response pulses gradually decrease in magnitude as the circuit stabilizes and the error is minimized. This error-proportional response is analogous to biological calibration processes such as temperature regulation, where the amount of energy consumed through sweating or shivering depends on the perceived temperature difference between the body and environment. The body then adjusts its reference temperature as it learns and becomes accustomed to the current ambient. Comparably, the simulated memristive circuit demonstrates energy-efficient learning through utilization of the passive non-volatile memory provided by the memristor and does not require a constant error signal be applied as with conventional control theory.


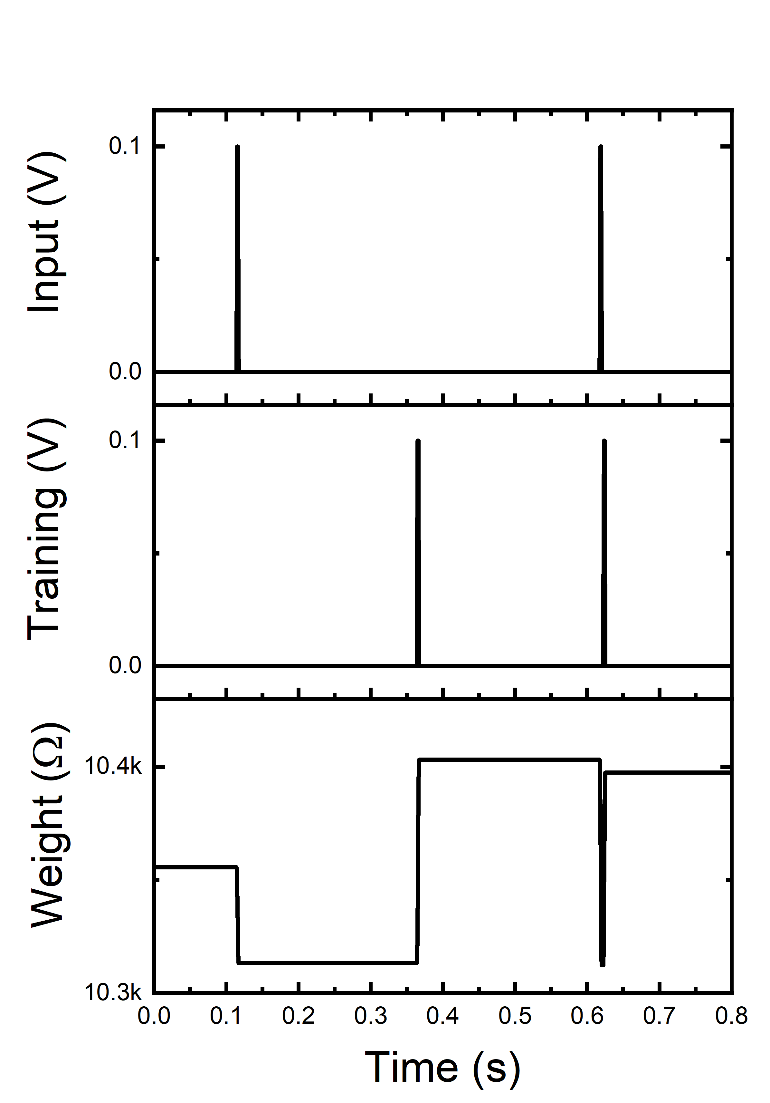


Supplementary Figure 7. SPICE transients demonstrating the STDP learning.

Supplementary Figure 7 shows the SPICE transients from an input (top), the system-wide training signal (middle), and an associated memristive weight (bottom) from a chronomemristive neural network used to classify images from the EMNIST dataset. One system-wide training pulse is given for every input image. Depending on whether the image contains the desired target, the response signal is pulsed within or outside of the chronomemristor’s temporal window. The first 100 mV input pulse shown in the top plot decreases the resistance state of the memristor (bottom plot) which is equivalent to increasing the strength of the synaptic connection via the memristor’s conductance. In this example, the first input image does not contain the desired target character. Thus, the response training pulse is timed to be outside the temporal window for STDP by delaying the training pulse (middle plot) from the input pulse by 250 ms. The first response pulse thus programs the memristor to a resistance state that is higher than the initial value before the first input pulse, resulting in a net increase in the resistance of the memristor, or effectively decreasing the memristive weight.

Similarly, the second input pulse temporarily strengthens the synaptic connection by increasing the conductance of the memristor. However, since the target is detected in the second input image, the response pulse is timed within the temporal window for STDP by delaying the training pulse from the input pulse by only 5 ms. As seen in Supplementary Figure 7 (bottom plot at ~0.6 s), the post/training pulse is desensitized and the memristive weight maintains a slightly higher conductance than before the pre/post (input-training) pulse cycle. Thus, training via the biological learning rule of STDP is verified within the architecture, where input images containing the target strengthen the synaptic connection while images without the target result in a weakened connection.
